# Bioinformatics Analysis Reveals Microrchidia Family Genes as the Prognostic and Therapeutic Markers for Colorectal Cancer

**DOI:** 10.2174/0118715303367767241231113110

**Published:** 2025-01-13

**Authors:** Binghui Liu, Lingbin Chen, Hui Chen, Juhua Pan, Changfa Yu

**Affiliations:** 1 Department of Pathology, Taizhou First People’s Hospital, Huangyan Hospital of Wenzhou Medical University, Taizhou, Zhejiang, China;; 2 Department of Laboratory Medicine, Taizhou First People’s Hospital, Huangyan Hospital of Wenzhou Medical University, Taizhou, Zhejiang, China

**Keywords:** MORC4, CRC, TCGA, tumor immunity, prognosis, cell invasion

## Abstract

**Aim:**

The aim of this study is to examine the role of the microrchidia (MORC) family, a group of chromatin remodeling proteins, as the therapeutic and prognostic markers for colorectal cancer (CRC).

**Background:**

MORC protein family genes are a highly conserved nucleoprotein superfamily whose members share a common domain but have distinct biological functions. Previous studies have analyzed the roles of MORCs as epigenetic regulators and chromatin remodulators; however, the involvement of MORCs in the development and pathogenesis of CRC was less examined.

**Objective:**

The current work examined the role of the MORCs as the therapeutic and prognostic markers for CRC.

**Methods:**

The expressions and prognostic significance of MORC family genes in CRC were explored. The role of these genes in tumor immunity was comprehensively analyzed in terms of their functions in immune cell infiltration, tumor microenvironment (TME), and their interaction with immune regulatory genes such as immunosuppressive genes, immune checkpoints and immunostimulatory genes. The relations between MORC family genes, tumor mutation burden (TMB), DNA, mismatch repair (MMR), RNA methylation, microsatellite instability (MSI), and drug sensitivity were investigated using the R statistical software. The expressions of MORC4 in 150 CRC tissues and 60 paracancer tissues were detected by immunohistochemical method. CRC cell proliferation, migration, and invasion were measured by cell counting kit-8 (CCK-8), scratch assay, and transwell cell invasion assay.

**Results:**

The expressions of MORC2 and MORC4 were significantly upregulated, whereas those of MORC1 and MORC3 were noticeably downregulated in CRC in comparison to their expressions in normal colorectal mucosal tissues. Patients with high-expressed MORC2 showed a more unfavorable prognosis than those with a low MORC2 level. Functional annotation analysis identified 100 MORC family genes with the most significant negative or positive correlations to diabetic cardiomyopathy, amyotrophic lateral sclerosis, oxidative phosphorylation, Huntington’s disease, thermogenesis, Parkinson’s disease, olfactory transduction, Alzheimer’s disease, prion disease. MORC3 expression was positively correlated with Stromal score, Immune score and ESTIMATE score, while MORC2 expression was negatively related to the three scores in CRC, these correlations were not statistically significant. Additionally, the MORC family genes were significantly positively correlated with tumor-infiltrating immune cells such as T helper cells and exhibited close relations to some immunosuppressive genes such as CXCR4 and PVR, immunostimulatory genes such as TGFBR1, KDR, and CD160 as well as some immune checkpoint genes. It was found that the expressions of some members of MORC family genes were positively correlated with DNA methylation, MSI, TMB, MMRs, and drug sensitivity in CRC and that the mRNA and protein levels of MORC4 were remarkably upregulated in CRC tissues than in adjacent normal tissues (*P*<0.05). In the MORC4 knockdown group, DLD-1 cell proliferation was more inhibited than in the negative control (NC) and siRNA groups (*P*<0.05). Furthermore, the migratory capacity of DLD-1 cells and the number of cells crossing the basement membrane in the MORC4 knockdown group were reduced compared to the NC and siRNA groups (all *P*<0.05).

**Conclusion:**

The expressions of MORC family genes were significantly different in CRC samples, which was related to the immune cell infiltration and prognosis of CRC. Thus, the MORC family genes were considered as markers for indicating the clinical immunotherapy and prognostic outcome of CRC.

## 1 INTRODUCTION

Colorectal cancer (CRC) is a frequently detected tumor worldwide [1-3]. In the last decade, the morbidity and mortality of CRC have increased rapidly in developing countries [4-6], including China [7]. Early diagnosis of CRC not only improves patient survival and life quality, but also reduces the difficulty of surgery and follow-up treatment (*e.g.* radiotherapy, *etc.*). However, the sensitivity, specificity, and invasiveness of early screening methods still need to be improved through the discovery of novel diagnostic and prognostic indicators, which will also contribute to the understanding of the pathogenesis of CRC and the development of targeted therapeutic drugs.

As a highly conserved nuclear protein superfamily, the members of microconidia (MORC) protein family have three signature structural domains, specifically, N-terminal conserved MutL-type-ATPase structural domain involved in DNA damage repair and regulation of transcription, intermediate CW-type zinc-finger structural domain involved in chromatin regulation by recognizing histone 3 lysine 4-methylated important histones, and the C-terminal convoluted helix structural domain crucial for protein interactions and protein subcellular localization [8-10]. Currently, MORC1, MORC2, MORC3, and MORC4 are the four members of the MORC family discovered in mammals [11]. Members of the MORC family are considered oncogenes overexpression is associated with higher malignancy and worse overall survival in various types of cancers, including liver cancer and breast cancer [12, 13]. Recent studies have shown that MORC family facilitates the development and spread of lung tumors in various lung cancer cell lines through activating the Wnt/β-catenin signaling pathway to enhance the recruitment of tumor-associated macrophages [14]. However, the molecular mechanisms underlying the MORC family as a promising target for CRC remain unclear.

This study performed bioinformatics analyses to explore the biological functions and prognostic value of MORC family genes in CRC as well as the relationship between MORC family genes and the TME. According to our findings, the MORC family genes can serve as the candidate diagnostic and prognostic biomarkers for CRC, and MORC4, in particular, was confirmed as a potential anti-CRC target.

## 2 MATERIALS AND METHODS

### 2.1 Expression Patterns of MORC Family Genes in CRC and Pan-Cancer

The survival data, expression profiles, and phenotypic information of the MORC family genes from tumor and non- tumor samples were acquired from The Cancer Genome Atlas (TCGA) database (https//portal.gdc.cancer.gov/). Additionally, demographics, tumor-related data and follow-up information of patients with the following cancers were collected: sarcoma (SARC), lung adenocarcinoma (LUAD), mesothelioma (MESO), hepatocellular carcinoma (LIHC), low- grade glioma of the brain (LGG), cell tumor (GBM), renal smoky cell carcinoma (KICH), prostate adenocarcinoma (PRAD), acute myeloid leukemia (LAML), ovarian cancer (OVC), renal clear cell carcinoma (KIRC), ovarian plasma cystadenocarcinoma (OV), cutaneous melanoma (SKCM), pancreatic adenocarcinoma (PAAD), testicular adenocarcinoma (TGCT), thymic adenocarcinoma (THYM), renal papillary cell carcinoma (KIRP), gastric adenocarcinoma (STAD), thyroid adenocarcinoma (THCA), uveal melanoma (UVM), head and neck squamous cell carcinoma (HNSC), lung squamous cell carcinoma (LUSC), pheochromocytoma and paraganglioma (PCPG), uterine sarcoma (UCS), and endometrial cancer (UCEC). The RNA-seq data were converted into the TPM format and log2 was transformed for subsequent analyses. Expression differences of genes in different cancer and corresponding normal tissue samples were compared using Mann-Whitney U test.

Subsequently, the mRNA expressions of MORC family genes in 50 normal colorectal tissues and 619 CRC tissues were detected. The mRNA expressions of MORC family genes in 50 CRC samples and corresponding non-carcinoma colorectal tissues were compared with Student's t-test, whereas those between unpaired samples were analyzed with Mann-Whitney U test. The R package was adopted to plot the receiver operating characteristic (ROC) curves to differentiate patients with high and low expressions of MORC family genes according to the median expression values of the genes. Next, the differentiation accuracy was assessed by the area under curve (AUC), with AUC value >0.90, AUC between 0.71-0.90, and AUC between 0.50-0.70 denoting high, intermediate, and poor diagnostic performance, respectively.

### 2.2 Association Between MORC Family Genes and Clinicopathological Features

The median RNA levels of the MORC family genes in CRC samples served as the cutoff to classify the CRC patients into high- and low-expression groups. Using the level of lymphatic invasion, perineural invasion, and presence of colon polyps as independent variables, the R “glm” function was used to conduct one-way logistic regression to analyze the relation between high and low RNA expressions of the MORC family genes and clinicopathological features of CRC patients (including age, TNM classification, clinical stage). Here, samples without available clinical data were excluded. Additionally, the associations between MORC family gene expressions and clinical stage were analyzed and visualized into box plots using the ggplot package of R statistical software. The correlations between the MORC gene family members were evaluated based on Pearson correlation coefficient, and the r-value and *p*-value of MORC family genes in CRC were calculated. Subsequently, the correlation heatmap of gene expressions was visualized by the ggplot2 package in R.

### 2.3 Prognostic Assessment of MORC Family Genes in CRC

Samples in the TCGA dataset without complete clinical data were removed. Combined with the preprocessed TCGA data, the CRC samples were classified into low- and high-expression groups by the median MORC family gene expressions. Next, “survival” package in R was adopted to perform survival analysis to evaluate the relations between the MORC family gene expressions, overall survival (OS), progression-free survival (PFS), disease-specific survival (DSS) (cutoff value of *p*<0.05), followed by visualizing the results in the Kaplan-XLR software. The relationship between MORC family genes, clinical parameters, and the OS of CRC patients was analyzed by univariate and multivariate Cox proportional risk regression, and the ggplot2 package was used to generate forest plots. Column line plots for 1-, 3-, 5- and year OS predicted for the patients in the TCGA-CRC dataset were developed. Subsequently, calibration curves were plotted by rms (version 6.2-0) and, survival packages and evaluated by comparing the predicted rates and actual rates.

### 2.4 Co-Expression Genes and Enrichment of MORC Family Genes

Using “DESeq2” (1.26.0) package in R, differentially expressed genes (DEGs) with significantly abnormal expressions in CRC and non-carcinoma tissues in the TCGA-CRC dataset were screened to identify those most closely related to the MORC genes (*p* < 0.05 and |cor Pearson (r)| > 0.3) [15]. We found that 1,080 genes were associated with MORC1, 853 genes were associated with MORC2, 2,209 genes were associated with MORC3, and 272 genes were associated with MORC4. After removing the duplicates among these genes, a total of 1,988 genes closely related to the MORC family genes were subjected to gene set enrichment analysis (GSEA) to calculate the log2FC values of these genes using the cluster analyzer R package. Gene Ontology (GO) functional annotation and Kyoto Encyclopedia of Genes and Genomes (KEGG) analyses were performed on the top 100 and last 100 genes correlated with the MORC family genes. Pathways were obtained from KEGG, WikiPathways (WP), and Reactome (REAC) databases. Functional terms with adjusted *p*< 0.05, |NES| > 1, and FDR (or q- val) < 0.25 were considered as significantly enriched.

### 2.5 Relationship Between the Expressions of MORC Family Genes and Immunity

The TME of each CRC patient was analyzed by calculating Stromal score, Immune score and ESTIMATE score using ESTIMATE algorithm in R package [16]. Pearson correlation analysis was used to explore the relations between MORC family gene expressions, the three ESTIMATE scores, and the infiltration of 24 immune cell types in CRC tissues (under the threshold of |r|>0.4) were calculated by ssGSEA function in R package. The associations of MORC family genes with immunomodulatory genes, including immunosuppressants, immunostimulants, chemokines, and chemokine receptors, were further explored. The relationship between the expressions of MORC family genes and immune checkpoints was also examined by Pearson correlation analysis. The above results were visually displayed using the package “ggplot2”.

### 2.6 Correlation of the Expressions of MORC Family Genes with TMB, MSI and MMR

TMB was reported as a biomarker for indicating the therapeutic effect of immune checkpoint blockade (ICB) [17]. In this study, the single nucleotide variants (SNVs) from TCGA Genomic Data Commons (GDC) dataset were detected by MuTect2 software, and tmb function in R package was adopted to calculate the TMB of each tumor and integrated the TMB scores and the expression data of the MORC family genes. MSI, which refers to the deletion or insertion of repetitive units in tumor tissues relative to healthy human tissues, can change the length of a particular satellite. MMR is an intracellular DNA mismatch repair mechanism through which the loss-of-function of key genes disrupts the mechanism to repair DNA replication errors, leading to greater somatic mutations. The five known MMR genes are mutS homolog 6 (MSH6), mutS homolog 2 (MSH2), mutL homolog 1 (MLH1), epithelial cell adhesion molecule (EPCAM), PMS1 homolog 2 (PMS2). Differences in the MSI and TMB between high- and low-expressions of MORC family genes were compared by Wilcox rank-sum test, with the median value of gene expression as the cutoff.

### 2.7 Methylation Analysis of MORC Family Genes

DNA methylation is an epigenetic regulatory mechanism that refers to the reversible methylation at the fifth carbon atom of cytosine in CpG dinucleotides. Aberrant DNA methylation is regarded as a biomarker for cancer prognosis as it plays a crucial role in cancer development [18]. The promoter DNA methylation levels of the MORC family genes between non-carcinoma samples and CRC patients with different tumor stage and lymph node metastasis in various subgroups were collected from UALCAN database (http://ualcan.path.uab.edu/) and compared.

### 2.8 Single-Cell Functional Analysis of MORC Family Genes

CancerSEA database (http://biocc.hrbmu.edu.cn/CancerSEA/) is developed for the study of the functional states of cancer cells at a single-cell level and contains the data on stemness, proliferation, migration, invasion, apoptosis, DNA damage, angiogenesis, differentiation, cell cycle, inflammation, DNA repair, epithelial-mesenchymal transition (EMT), hypoxia, and silencing of 41,900 cancer cell lines across 14 functional states of individual cancer cells in 25 cancers [19]. This study used CancerSEA database to analyze the functional relevance of MORC family genes in CRC.

### 2.9 Drug Sensitivity of MORC Family Genes

GSCALite database is a platform for analyzing gene expression, pathway activity, methylation, SNVs, drug sensitivity [20]. The resistance of MORC family genes to various drugs for CRC therapies was analyzed. Next, the relationship between the expressions of MORC family genes and 265 small molecules collected from Genomics of Drug Sensitivity in Cancer (GDSC) database was studied based on Pearson correlation coefficient, with *p* < 0.05 denoting a statistical significance.

### 2.10 Immunohistochemistry and RT-qPCR Assays

The CRC tissue and paracancer tissue slices were dewaxed, gradient-hydrated, and antigenically repaired. The samples were blocked by 5% BSA at ambient temperature and then incubated with MORC4 primary antibodies (1:100; Abcam; UK) overnight. Next, the slices were washed and further probed with secondary antibodies for 1 hour (h) at ambient temperature. After discarding the secondary antibodies, DAB staining was performed by adding horseradish peroxidase. Next, the slices were incubated with hematoxylin, dehydrated with ethanol, treated with xylene, and finally sealed with a film until the MORC4 antibody was mostly localized in the cancer cell nuclei that were observed to be stained brownish-yellow under the microscope. Scoring was performed based on the porportion of stained cells, with 0-4 points indicating >5%, 6-25%, 26-50%, 51-75% and >75% positively stained cells, respectively. The cells were also scored according to staining intensity, with 0-3 points denoting non-positive intensity, light, medium, and strong staining, respectively. Thereafter, the results of these two scores were added to obtain the final score, with 0-1, 2-4, 6-8, and 9-12 points suggesting negative (-), weakly (+), moderately (++), and strongly positive (++++), respectively. Expression differences of MORC4 between CRC and paracancerous samples were compared by χ^2^ test, with *p* < 0.05 denoting a statistical significance.

Total RNAs were separated from CRC and paracancerous tissues by Trizol and reverse-transcribed to cDNA. QRT-PCR assay was carried out following the protocol of TB Green Premix Ex Taq^TM^ kit. Normal colorectal mucosal tissues adjacent to the cancer tissues were used as a reference. GAPDH was used as an endogenous control, and 2^-ΔΔ^ Ct value was calculated to indicate relative level of MORC4. The primer sequence of MORC4 was F:5ʹ-TGGATTGAGCACCAGACTGT-3ʹ and R:5ʹ-AACTGGCCTCTTTCTCCACA-3ʹ and the GAPGH internal reference primer sequence was F:5ʹ-GTCAACGGATTTGGTCTGTATT-3ʹ and R:5 ʹ-AGTCTTCTGGGGTGGCAGTGAT-3ʹ. SPSS 23.0 statistical software was employed for data analysis. Differences in the mRNA expression of MORC4 between two-group independent samples were compared by the t-test. *P* < 0.05 denoted a statistical significance. The Medical Ethics Committee of Taizhou First People’s Hospital (approval number: 2022-KY018-01) reviewed and approved the present work.

### 2.11 Cell Transfection and Grouping

Differences in the mRNA expression of MORC4 in CRC cell lines (HCT-15, SW480, SW620, DLD-1, and HCT116) and human colon epithelial cells (NCM460) were compared to select the CRC cell line with the largest relative expression as the transfected cells. Cells at the logarithmic phase were seeded into 6-well plates and cultured at 37°C with 5% CO_2_ to 70% cell fusion for cell transfection. Following the instructions of Lipofectamine 2000 reagent, siRNA (50 nmol) and Lipofectamine 2000 reagent (6 µL) were added into Opti-MEM medium (200 µL) to incubate the cells for 15 minutes (min). Next, the medium was added into the 6-well plate and supplemented with 2 mL of Opti-MEM. Approximately 6 h after the transfection, the complete medium was replaced with antibiotic-free medium. After 48 h, the cells were harvested for subsequent analyses and classified into blank control (without any reagent), negative control (transfected with siRNA), and knockdown (transfected with si-MORC4) groups.

### 2.12 CCK-8 Assay and Cell Scratch Invasion Test

The three groups of cells were digested, washed, adjusted to 5,000 cells/well and seeded into the 96-well plates (100 µL/well) to allow the cells to attach to the wall. Next, CCK-8 reagent (10 µL) was added into each well at 0, 24, 48 and 72 h for 2-h incubation. The absorbance was read at 450 nm with an enzyme labeling instrument.

To perform the cell scratch experiment, the cells were digested, washed, adjusted to 5,000 cells/well and inoculated into six-well plates. When the cells covered the well bottom, a 10 µL tip was applied to draw a horizontal line on the cells, which were then rinsed in sterile PBS three times to gently remove cell debris and then photographed (0 h). Next, the scratched cells were further incubated with culture medium, similarly washed and photographed, and further incubated for 48 h. Changes in scratches was analyzed with the ImageJ software. The wound healing rate (%) was calculated as:

Scratch healing rate (%) = [(0 h scratch area - 48 h scratch area)/0 h scratch area] × 100%.

For transwell cell invasion assay, firstly, the upper chamber was inserted into 24-well plates to incubate the above three groups of cells for 24 h, while fresh medium containing 10% FBS was added into the lower chamber. Next, the invading cells were fixed by 4% paraformaldehyde for 15 min, dyed using 0.1% crystal violet, and counted under a microscope [21].

## 3 RESULTS

### 3.1 The Expression Patterns of MORC Family Genes in CRC

**Fig. (2):**
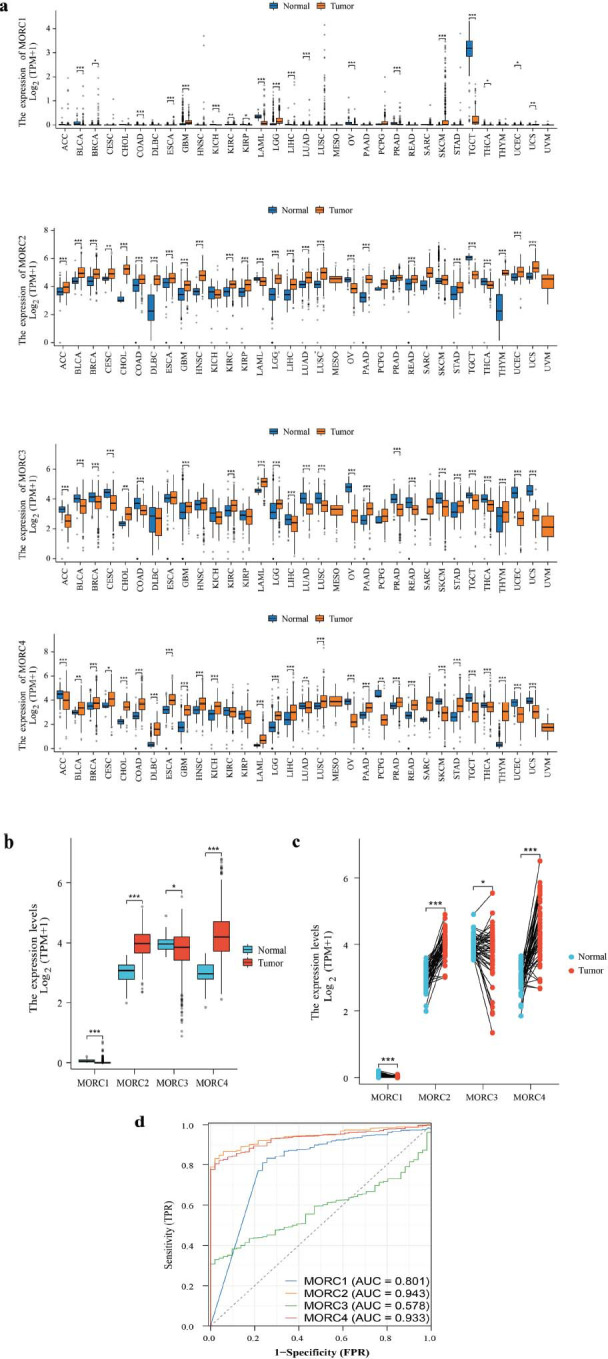
Expression of MORC family genes. (**a**) Expression of the mRNAs of MORC family genes in pan-cancer. (**b**) Expression levels of MORC family genes in non-paired tissues of colorectal cancer. (**c**) Expression levels of MORC family genes in CRC in matched tumor and normal tissues. (**d**) The predictive ability of MORC family genes for the diagnosis of malignant tumors in CRC. *p > 0.05, **p > 0.01, ***p > 0.001

Fig. (**1**) shows the study flowchart. MORC2 and MORC4 were upregulated in most tumors such as HNSC, READ, STAD, PRAD, LIHC, BLCA, COAD, ESCA, LUSC, and CHOL (Fig. **2a**). The expression of MORC2 in KICH and THCA and that of MORC4 in KIRP, PCPG, and UCEC was downregulated. The level of MORC1 was upregulated in KICH, BRCA, COAD, KIRP, PRAD, and READ but downregulated in UCEC, BLCA, and LIHC. MORC3 was upregulated in LIHC, CHOL, ESCA, KIRC, and STAD but downregulated in CESC, COAD, LUAD, and LUSC. Compared to healthy colorectal mucosal tissues, the expressions of MORC2 and MORC4 were higher and those of MORC1 and MORC3 were lower in CRC samples (both *P* < 0.05) (Figs. **2b** and **2c**). It could be observed that MORC1, MORC2, and MORC4 manifested a high diagnostic value for CRC, with an AUC of 0.801, 0.943, and 0.933, respectively, whereas the diagnostic accuracy of MORC3 was lower (AUC = 0.578) (Fig. **2d**).

### 3.2 Relationship Between the Expressions of MORC Family Genes and Clinicopathological Characteristics of CRC

Logistic regression was employed to analyze the relation between mRNA levels of MORC family genes and clinical features of CRC patients (Table **1**). It was observed that MORC2 was positively related to clinical stage (*P* = 0.005), tumor T-stage (*P* = 0.038), N-stage (*P* = 0.014), and M-stage (*P* = 0.023). Meanwhile, MORC3 expression was positively correlated with residual tumor (*P* = 0.007) and lymphatic invasion (*P* < 0.001), and MORC4 expression showed positive relation to N-stage (*P* = 0.001), M-stage (*P* = 0.015), and clinical stage (*P* < 0.001).

### 3.3 The Significance of MORC Family Genes in the Prognostic Evaluation of CRC

**Fig. (3):**
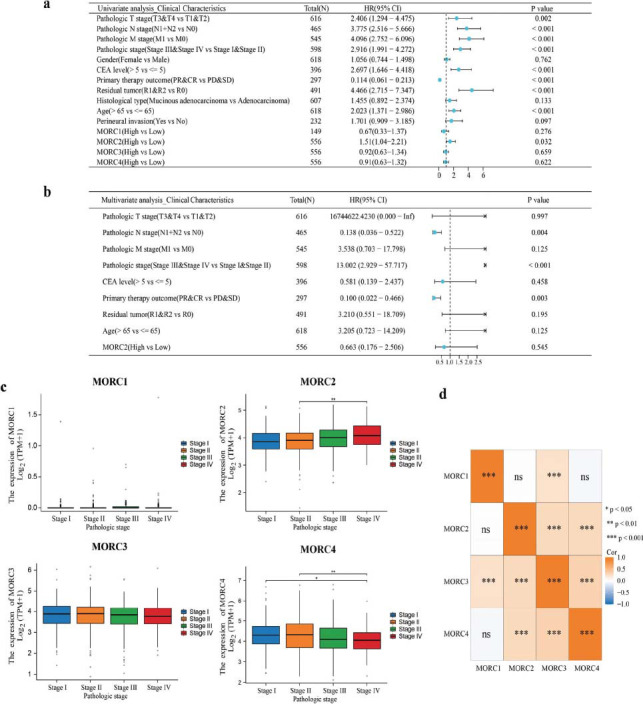
The relationship between each MORC family gene and clinical characteristics. (**a** and **b**) Univariate and multivariate Cox regression analyses were performed to determine the association between MORC family genes and clinical characteristics. (**c**) Expression of MORC family genes in CRC patients with different TNM stages. (**d**) A heat map of the correlation between MORC family genes in CRC; *p < 0.05, **p < 0.01, and ***p < 0.001.

**Fig. (4):**
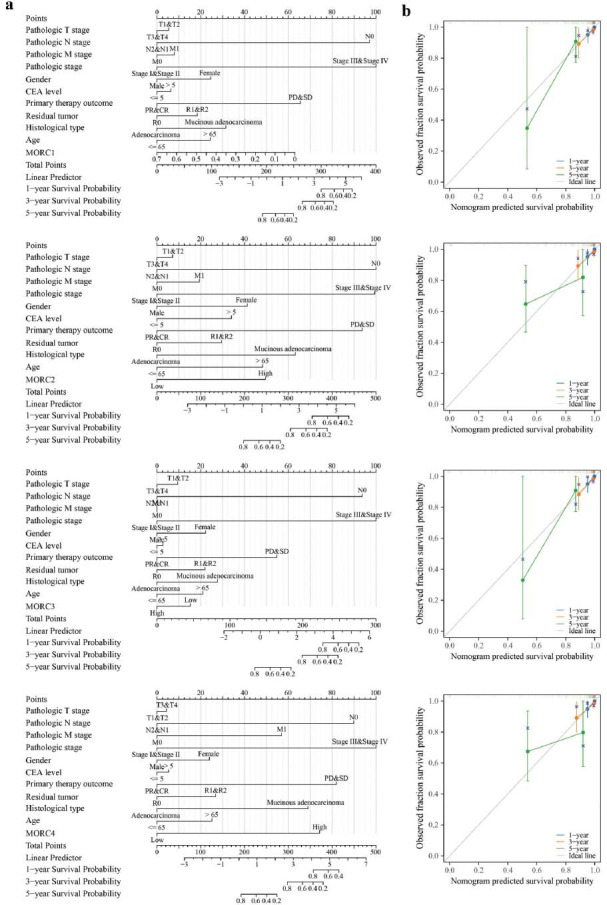
Nomogram plots and calibration plots. (**a**) A nomogram plot model for predicting the survival rate of colorectal cancer by combining MORC family genes with clinical pathological parameters. (**b**) A calibration curve of the nomogram plot model.

The relation between the expressions of MORC family genes and CRC prognosis was analyzed based on the CRC-related data from TCGA dataset. CRC patients with high- expressed MORC2 showed significantly worse OS, DSS and PFS than those with low-expressed MORC2 (*P* < 0.05) (Fig. **S1a**). Overexpressed MORC2 was closely related to dismal CRC prognosis but the exprssions of MORC1, MORC3, and MORC4 were irrelevant to OS, DSS, or PFS in CRC (Figs. **S1b**, **S3c**, and **S3d**). According to the univariate Cox proportional risk model, clinical stage, serum CEA level, tumor T-stage, N-stage, M-stage, clinical treatment effect, radiotherapy, age, and the expression of MORC2 were related to the OS rate of CRC patients. The results of the multivariate Cox proportional risk model showed that tumor N-stage, clinical stage, and treatment effect could independently indicate the prognostic outcomes of CRC patients (Figs. **3a** and **3b**). MORC2 expression was markedly elevated in CRC patients at stage IV than those at stage II (*P* < 0.05) (Figs. **3c** and **3d**), and MORC4 expression in CRC patients at stage I and stage II CRC was more upregulated than those at stage IV. Nonetheless, no significant relation between CRC pathological stages and the expressions of MORC1 and MORC3 was observed (*P* > 0.05). MORC1, MORC2 and MORC4 were not correlated with each other but the rest of each pair of MORC family genes were clsely positively related (*P* < 0.01). The survival of patients who had higher total ESTIMATE scores was worse. As shown by calibration curves, the MORC family genes performed well in predicting 1- and 3- year OS (Fig. **4**).

### 3.4 Biological Function and Enrichment Analysis of MORC Family Genes

**Fig. (5):**
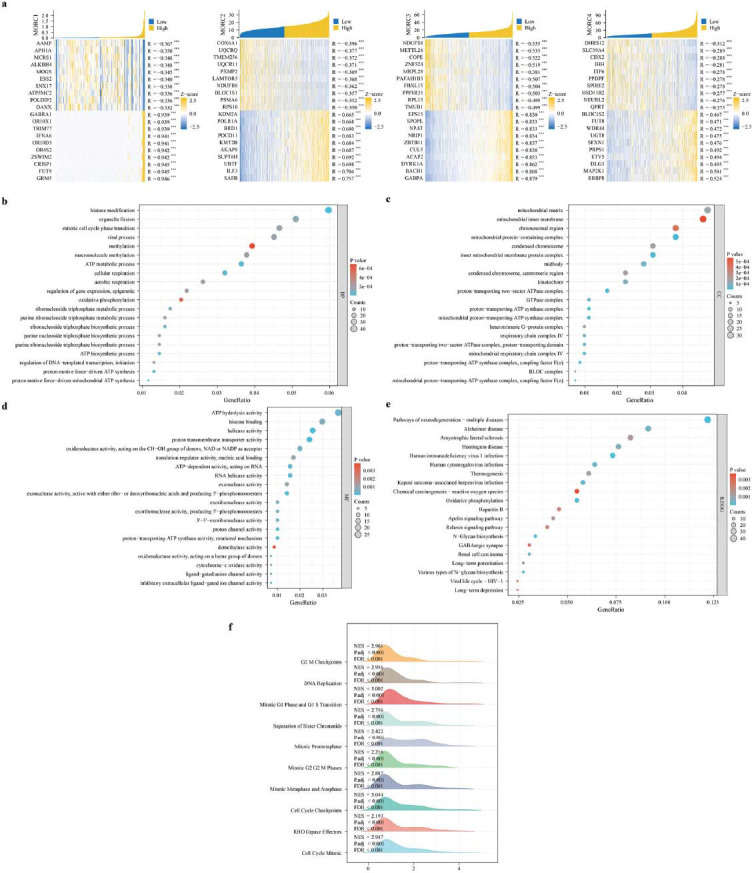
Functional analysis of MORC family genes in CRC. (**a**) The heatmaps illustrate the expression of the top 10 positively correlated genes and the top 10 negatively correlated genes with the genes of the MORC family. (**b**) GO_BP, (**c**) GO_CC, (**d**) GO_MF and (**e**) KEGG enrichment analyses of the top 100 genes positively and negatively correlated with the genes of the MORC family. (**f**) The mountain plot shows the results of gene set enrichment analysis (GSEA), which was performed based on the significantly correlated genes (correlation coefficient value |r| > 0.3; *P* > 0.05) in the MORC family.

The top 100 and last 100 genes in the TCGA database related to the MORC family genes were subjected to GO annotation (Fig. **5a**). The MORC family genes were related to histone modification, macromolecule methylation, protonmotive force-driven mitochondrial ATP synthesis, TP metabolic process, oxidative phosphorylation, protonmotive force-driven ATP synthesis histone modification, methylation, and ATP biosynthetic process in biological process (BP) term (Fig. **5b**). In cellular component (CC) term, the MORC family genes were associated with proton-transporting two-sector ATPase complex, mitochondrial protein-containing complex, proton-transporting ATP synthase complex, inner mitochondrial membrane protein complex, coupling factor F(o), mitochondrial proton-transporting ATP synthase complex, and mitochondrial respiratory chain complex IV (Fig. **5c**). In molecular function (MF) terms, the MORC family genes were associated with proton transmembrane transporter activity, proton-transporting ATP synthase activity rotational mechanism, ATP hydrolysis activity, oxidoreductase activity, cytochrome-c oxidase activity (Fig. **5d**). KEGG enrichment analysis revealed that the MORC family genes were enriched to N-Glycan biosynthesis, human immunodeficiency virus 1 infection, oxidative phosphorylation, renal cell carcinoma, and pathways of neurodegeneration-multiple diseases (Fig. **5e**). GSEA revealed that these genes were significantly associated with pathways such as ECM regulators, G2-M checkpoint, PLK1 pathway, cytokine-cytokine receptor, cell cycle checkpoints (Fig. **5f**).

### 3.5 Association Between MORC Family Genes and Immune Infiltration in CRC

The relation between the MORC family genes, TME, tumor immune-infiltrating cells, and immune-related genes was analyzed. ESTIMATE method showed that MORC1 expression was positively related to Stromal score (R = 0.127, *P* < 0.05), and that MORC3 expression was positively related to Stromal score (R = 0.213, *P* < 0.05) and ESTIMATE score (R = 0.260, *P* < 0.05). However, the expressions of MORC2 and MORC4 had low or even no correlation with the TME in CRC (Fig. **S2**). As shown by Pearson correlation analysis, MORC family genes were significantly related to immune cell infiltration, including TCM, T helper cells, Th17 cells, and CD56dimNK cells (Fig. **S3**).

**Fig. (6):**
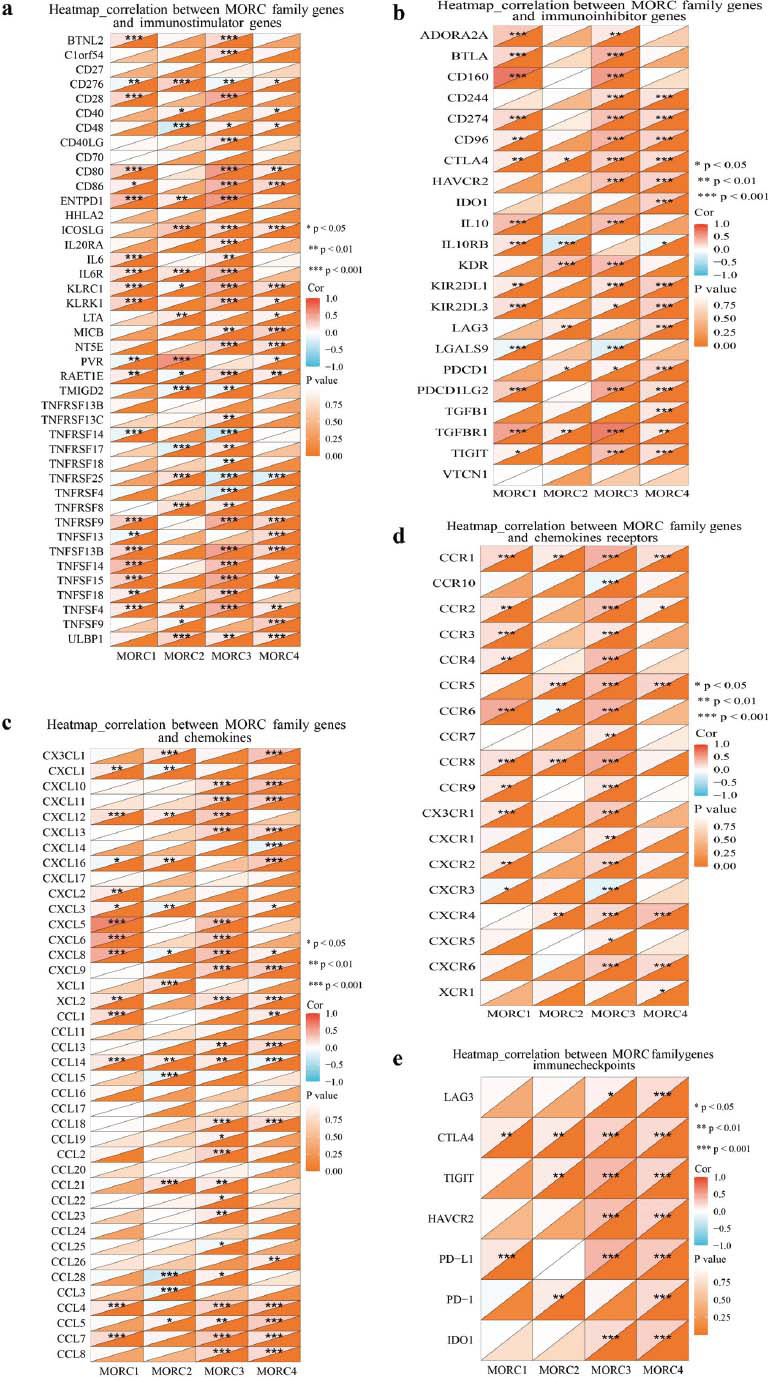
(**a** and **b**) Correlation between MORC family genes and immunostimulatory/immunoinhibitory genes in CRC. (**c**-**e**) Correlation between MORC family genes and chemokines/chemokine receptors/immune checkpoints in CRC. *p > 0.05, **p > 0.01, ***p > 0.001.

Analysis of the relation between MORC family genes and immunomodulatory genes showed that MORC3 and MORC4 levels were positively related to immunoregulatory genes such as MHC, immunostimulator genes, immune inhibitory genes, chemokines, and chemokine receptors in CRC (Figs. **6a**-**d**). Additionally, MORC3 and MORC4 were positively related to immune checkpoint levels, including PD-L1, CTLA4, TIGIT, and HAVCR2 (Fig. **6e**).

### 3.6 Correlation Between MORC Family Genes with TMB, MSI, and MMR Genes

By exploring the correlation between the MORC family genes and TMB, MSI, and MMR genes closely related to the efficacy of immune checkpoint inhibitors (ICIs), it was observed that TMB and MSI were significantly different between cells with high and low MORC4 expression in CRC (*P* < 0.05) (Figs. **S4a** and **S4b**). Furthermore, MSH2, MSH6, and PMS2 were positively related to MORC4 expression (Fig. **S4c**) but was not significantly related to MORC1, MORC2, or MORC3.

### 3.7 Relation Between MORC Family Genes and Methylation

**Fig. (7):**
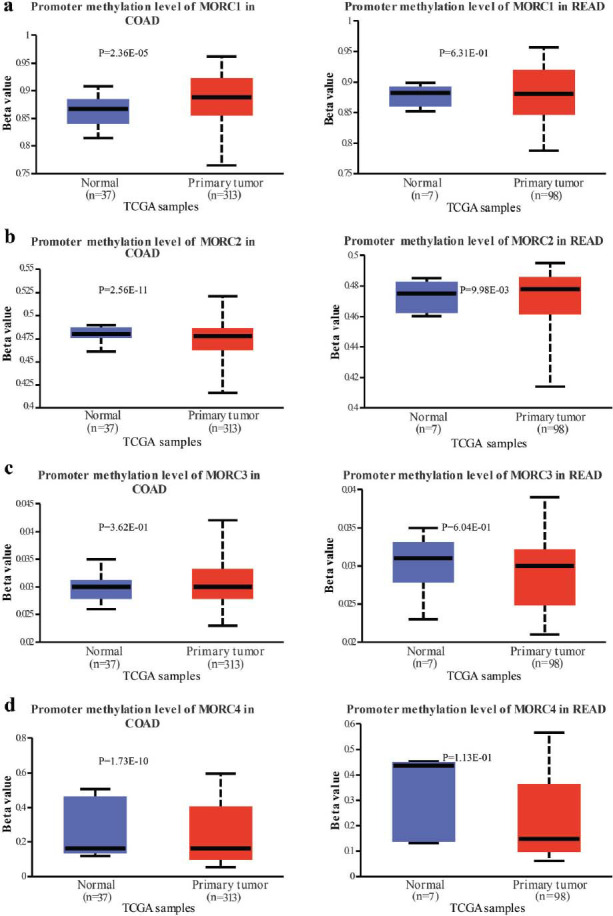
The relationship of MORC family genes with methylation and methyltransferase in CRC. The promoter methylation level of MORC1 (**a**), MORC2 (**b**), MORC3 (**c**), and MORC4 (**d**) in CRC.

**Fig. (8):**
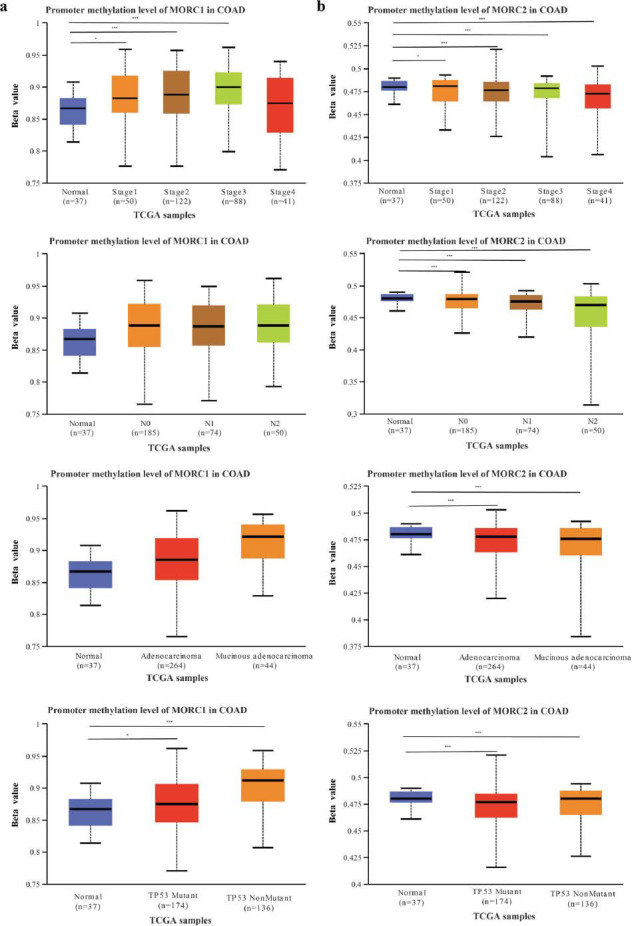
Methylation level of MORC1 (**a**) and MORC2 (**b**) related to different clinicopathological features of colon cancer (* *P* < 0.05, and *** *P* < 0.001).

Based on UALCAN database, it was found that the methylation level of MORC1 was significantly elevated in CRC tissues than in normal tissues, whereas that of MORC2, MORC3 and MORC4 were significantly lower in CRC tissues (*P* < 0.05) (Figs. **7a**-**7d**). Additionally, the MORC2 promoter methylation was markedly increased in CRC tissues (*P* < 0.05), but the methylation of MORC1, MORC3, and MORC4 was significantly lower in the tumor tissues than in negative controls (Fig. **7b**). Next, we examined the relations between the methylation levels of MORC1 and MORC2 and clinicopathological features in CRC. The methylation levels of MORC1 in stages I, II, III, N0, N1, and N2, different molecular subtypes of colon cancer, TP53 mutation, TP53 wild-type, and normal colon tissues were all significantly different (*P* < 0.05) (Fig. **8a**). Similarly, MORC2 methylation levels in stage, lymph node metastasis, TP53 mutation were remarkably different between different molecular subtypes in colon cancer and healthy colon samples (*P* < 0.05) (Fig. **8b**). However, the methylation of MORC3 and MORC4 and the clinicopathologic features of different subtypes of CRC were not significantly different (Fig. **S5**).

### 3.8 Single-Cell Functional Analysis of MORC Family Genes

**Fig. (9):**
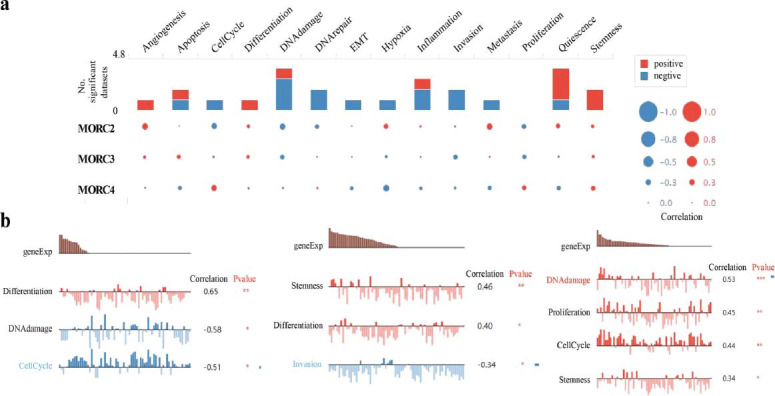
The function of MORC family genes in CRC was determined by single-cell functional analysis in the CancerSEA database. (**a**) The functional status of MORC family genes in CRC. (**b**) Correlation analysis between the functional status and MORC family genes in CRC; ** *p* < 0.01, and *** *p* < 0.001.

Considering the presence of intra-tumor heterogeneity in cancer cells, single-cell RNA-seq was applied to reveal the interrelationships and cell differences in the same tumor at the single-cell level. According to the results from CancerSEA database, the expression of MORC1 was irrelevant to functional status of CRC cells; MORC2 was positively related to differentiation (R = 0.65, *P* < 0. 01) and negatively related to DNA damage and cell cycle (R = -0.58 and -0.51, respectively, both *P* < 0. 05); MORC3 was positively correlated with differentiation and stemness (R = 0.46 and 0.40, separately, both *P* < 0. 05) but negatively correlated with invasion (R = -0.34, *P* < 0. 05); MORC4 expression was positively related to DNA damage, proliferation, cell cycle, and stemness (R = 0. 53, 0. 45, 0.44, and 0.34, separately, *P* < 0. 05) (Fig. **9**).

### 3.9 Drug Sensitivity Analysis of the MORC Family Genes

**Fig. (10):**
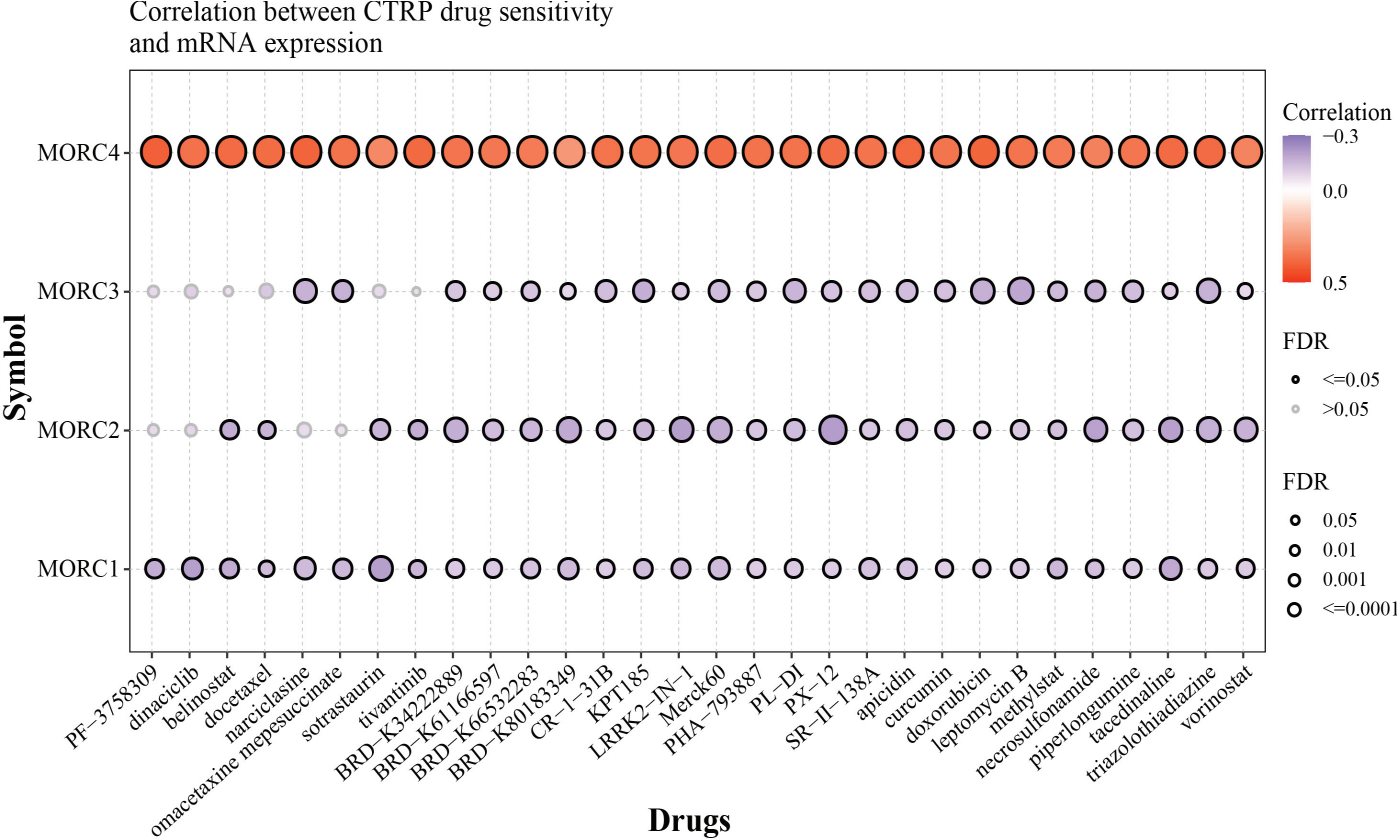
The association between the expression of MORC family genes and drug sensitivity was assessed by using the GSCALite database.

The relation between the MORC family genes and different drug targets was analyzed. Specifically, MORC1 can be inhibited by small-molecule drugs such as BRD-K34222889, BRD-K30748066, dinaciclib, sotrastaurin, and tacedinaline; MORC2 can be inhibited by tacedinaline, PX-12, and LRRK2-IN-1; MORC3 can be inhibited by leptomycin B and triazolothiadiazine. MORC4 was closely related to the activity of most drugs, and patients with a high expression of MORC4 were resistant to these drugs (Fig. **10**).

### 3.10 Validation of the Expression of MORC4 and Its Potential Biological Functions in CRC

**Fig. (11):**
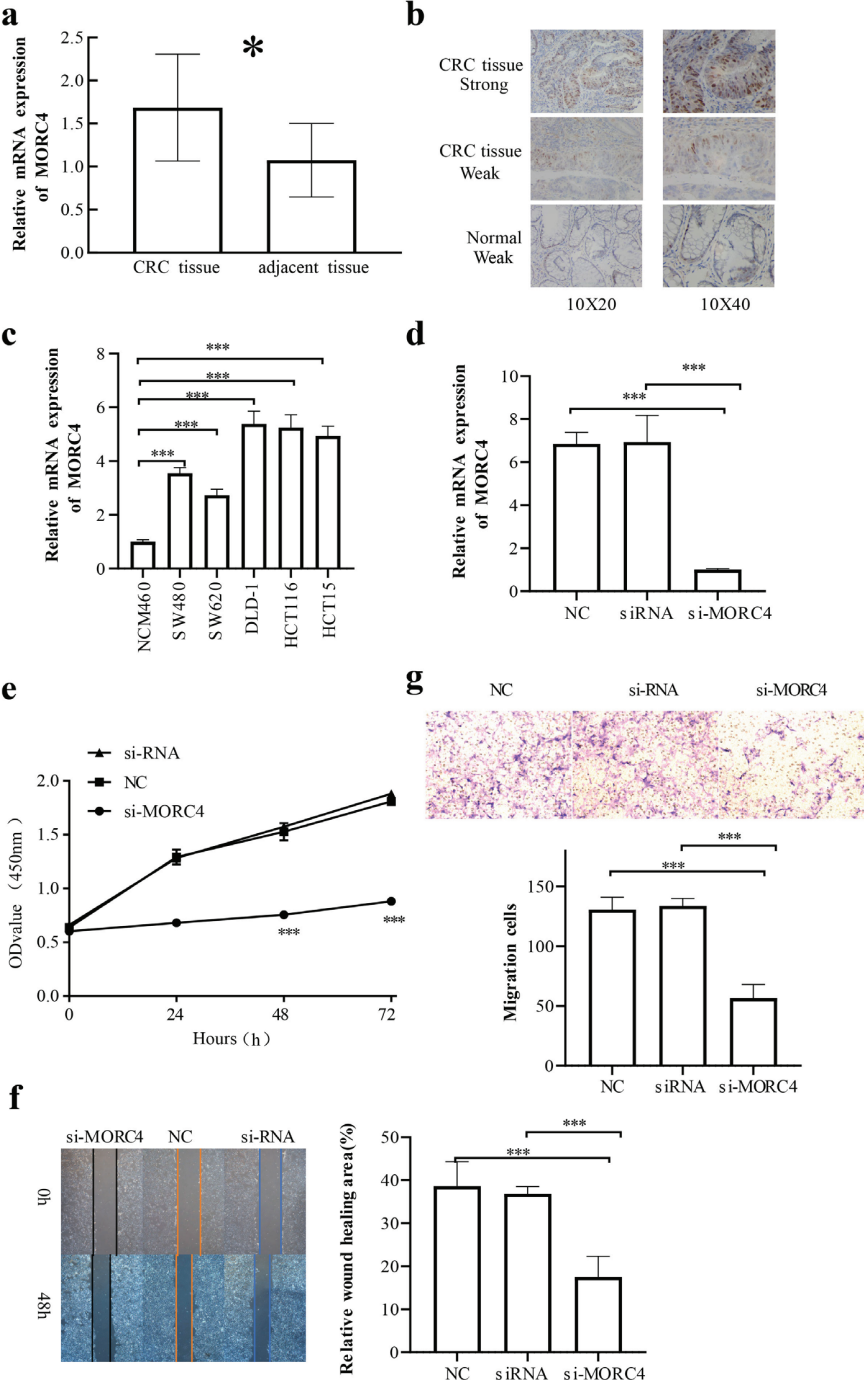
MORC4 expression in tumors and normal tissues was determined by immunohistochemistry and RT-qPCR assays in CRC. The results showed that MORC4 promotes the growth and metastasis of CRC cells *in vitro*. (**a**) The qRT-PCR analysis of cancer tissue and adjacent normal tissue samples from CRC patients. (**b**) Representative images of the expression of the MORC4 protein in paraffin-embedded CRC tissue and adjacent normal tissue. (**c**) The mRNA levels of MORC4 were analyzed in CRC cell lines (DLD-1, HCT116, HCT15, SW620, and SW480) and a normal human colon epithelial cell line (NCM460). (**d**) The MORC4 mRNA level decreased considerably after si-MORC4 transfection compared to its level in the control groups (NC, si-RNA group) in DLD-1 cells, as determined by RT-qPCR. (**e**) The CCK-8 assay was performed to evaluate the proliferation of DLD-1 cells after si-MORC4 transfection. (**f**) Wound healing assays were performed in DLD-1 cells transfected with NC, siRNA, or MORC4 siRNA. (**g**) Cell migration assays were conducted using DLD-1 cells transfected with NC, siRNA, or MORC4 siRNA; ***P < 0.001.

The mRNA expression of MORC4 was markedly elevated in CRC tissues than in paracancerous tissues (*P* < 0.01) (Fig. **11a**). Immunohistochemical staining assays showed that cell nuclei were brownish-yellow and more distributed in CRC tissues than in paracancerous tissues (Fig. **11b**). The expression of MORC4 protein in the CRC tissues reached 62.00% (93 out of 150), showing a significantly upregulating trend (11.67%; 7/60) than in paracancerous tissues (*P* < 0.05). The MORC4 expression in CRC tissues derived from patients with different genders, ages, tumor locations (left or right side), tumor diameters, and T-stage showed no significant difference (*P* > 0.05). However, the MORC4 expression was significantly different in the tissues derived from CRC patients with distant metastasis, different degrees of differentiation, lymph node metastasis, serum CEA level, and TNM stage (all *P* < 0.05) (Table **2**).

According to the qRT-PCR assay, the mRNA expression of MORC4 in SW480, SW620, DLD-1, HCT116, and HCT15 was markedly elevated than in NCM460 (all *P* < 0.05). Among all experimental cell lines, MORC4 was the most upregulated in DLD-1 cells. The mRNA expression of MORC4 in the knockdown group of cells was markedly inhibited than in NC and blank groups (*P* < 0.05) (Fig. **11c**). The cellular absorbance at 450 nm of the knockdown group was remarkably lower than NC and blank groups 24, 48, and 72 h after incubation (Fig. **11d**). The above findings indicated that the CRC cell proliferation was remarkably suppressed in the knockdown group (*P* < 0.05) (Fig. **11e**). The scratch healing rate in the knockdown group was also more noticeably inhibited than in NC and blank groups (all *P* < 0.05) (Fig. **11f**). The number of invasive cells in knockdown group apparently decreased when compared to NC and blank groups (all *P* < 0.05) (Fig. **11g**).

## 4 DISCUSSION

CRC has a high death rate [22]. Complications after CRC surgery, such as surgical site infection (SSI) and sepsis will seriously affect patients' prognosis and quality of life [23, 24]. SSI is one of the most common postoperative complications in CRC patients, and its occurrence will slow down postoperative recovery or even threaten the life of patients [25]. Sepsis, a systemic infectious response, is often accompanied by a significant inflammatory response that may lead to multiple organ failure [26]. In recent years, biomarker studies have contributed to the prediction and early diagnosis of postoperative complications of CRC. Butyrylcholinesterase (BChE) is a novel biomarker for a variety of disease states [27]. The relationship between BChE, inflammatory response, infection and liver function has been gradually recognized [28, 29]. Studies have shown that BChE has a significant impact on gastric cancer development and progression and is therefore considered a biomarker for detecting gastric cancer [30]. Similarly, discovering novel prognostic molecular markers for CRC can also contribute to the early screening and prediction of postoperative complications.

The MORC family is a highly conserved nuclear protein superfamily in humans, mice, nematodes, plants (*e.g.,* *Arabidopsis thaliana*), and slime molds [31]. Overexpressed MORC4 accelerates the development of CRC by controlling PCGF1/CDKN1A signaling [32]. Zhao *et al.* found that upregulated MORC2 is indicative of a poor prognosis in patients with colon adenocarcinoma and that MORC2 expression is related to immune cell infiltration, such as NK cells [33]. Similarly, this study observed that the expressions of MORC2 and MORC4 were remarkably upregulated, while MORC1 and MORC3 were downregulated in CRC compared to normal tissues. MORC2 overexpression was predictive of a worse prognosis of CRC and was significantly positively related to NK cell infiltration. Notably, we observed that all the MORC family genes were significantly and positively related to T helper cell infiltration. T helper cells play a crucial role in the immune system of the body by secreting cytokines that help activate or coordinate the functions of other immune cells [34]. T helper cell subsets are also involved in CRC progression [35]. Hence, these findings suggested that the MORC family genes were also closely involved in the regulation of immune responses and the development of CRC.

TMB and MSI can indicate the immunotherapeutic response in different types of tumors, therefore, measuring TMB has the potential to predict tumor susceptibility, immunotherapies and prognosis for cancer patients. Highly mutated tumors contain a high neoantigen load that enables the tumors to become immunogenic and responsive to immunotherapy [36]. The KEYNOTE-158 study applied pembrolizumab to treat non-CRC patients and found a remarkably higher objective remission rate in high TMB patients than those with a low TMB (29% *vs.* 6%) [37]. MSI is a result of code-shifting mutations in DNA pairing due to functional defects in MMR system. MSI is classified into microsatellite instability high-frequency (MSI-H), microsatellite instability low-frequency (MSI-L), and microsatellite stability (MSS). MSI has recently become the basis of stratification for routine treatment of CRC and a prognostic indicator for immunocheckpoint therapy. According to our findings, MORC4 expression was positively correlated with TMB and the expressions of MSH2, MSH6, and PMS2 in CRC, while MORC1, MORC2, and MORC3 were not correlated with TMB or MSI. These findings indicated that a high expression of MORC4 in CRC could indicate greater benefits from immunotherapy. Hence, MORC4 was considered a novel candidate therapeutic target for anti-CRC immunotherapy.

DNA methylation refers to the formation of 5-methylcytosine through selectively adding one methyl group into the fifth carbon atom of cytosine at the DNA sequence-specific site (CpG dinucleotide) under the action of methyltransferases (DNA methyltransferases (DNMTs), thereby playing an important role in controlling cell nuclear structure and gene expression [38, 39]. The present study observed that compared to normal tissues, the promoter methylation levels of MORC1 and MORC2 were remarkably higher in COAD and READ, respectively. DNA methylation of the MORC1 promoter region is closely associated with depression scale scores and is regarded as a stress-sensitive gene for depression [40]. Wang *et al.* found that MORC2 enhances the stemness of cancer cells and tumor formation by silencing DNA methylation-dependent Hippo signaling, suggesting that it may serve as a potential target for treating cancers [41]. These results showed an epigenetic regulatory mechanism of MORC family genes in CRC progression. In conclusion, these differential methylation patterns may provide a basis for developing more effective diagnostic and prognostic markers for CRC.

CancerSEA was used for pan-cancer functional analysis of MORC family genes. According to our single-cell functional analysis, the levels of MORC family genes were positively related to cell differentiation, stemness, DNA damage, and cell cycle. Some studies showed that upregulated MORC2 is predictive of a worse prognosis of colon cancer [33]. MORC4 has a high expression in breast cancer cells and tissues, and knocking down MORC4 suppresses breast cancer cell activity and promotes their apoptosis [42]. According to our findings, MORC4 was remarkably high-expressed in CRC tissues and cells, indicating the potential role of MORC4 as an oncogene of CRC. As shown by further analysis of the relation between MORC4 and clinicopathological factors, the MORC4 expression was not significantly different in CRC tissues derived from patients with different genders, ages, tumor locations (left and right side), tumor diameters, or T-stage. However, MORC4 expression showed statistically significant difference in CRC tissues from patients with different TNM stage, serum CEA levels, with or without distant metastasis and lymph node metastasis. We also found that the OS of CRC patients with high-expressed MORC4 was noticeably worse than those with a low-expressed MORC4, indicating the potential of MORC4 in determining the malignancy degree and prognosis of CRC. Knocking down MORC4 in CRC cells suppressed their proliferation, invasion and migration, which confirmed that MORC4 could be a candidate target for treating CRC.

Analysis on the relation between MORC family genes and anticancer drug sensitivity showed that MORC1 can be suppressed by some small-molecule drugs such as BRD-K34222889, BRD-K30748066, dinaciclib, sotrastaurin, and tacedinaline. Additionally, MORC2 can be inhibited by tacedinaline, PX-12, and LRRK2-IN-1, and MORC3 can be inhibited by leptomycin B and triazolothiadiazine. Noticeably, MORC4 showed a significant positive relation to the activities of most drugs that are able to suppress tumor development. However, there were some limitations to be noted, for example, most of the current analyses were based on bioinformatics, and further *in vivo* animal models are required to validate the functions of MORC family genes. In addition, the sample source should be expanded to include more clinical samples and patient data from different races and regions to improve the generalizability and applicability of our findings. Finally, future studies are encouraged to examine the relationship between MORC family genes and cancer-related pathways and to explore the potential synergistic mechanisms.

## CONCLUSION

This research systematically analyzed the expressions and functions of MORC family genes and their correlation with prognosisin CRC. It was found that the expressions of MORC2 and MORC4 genes were upregulated in CRC, and that high-expressed MORC4 was associated with a poor prognosis of CRC patients. In addition, the correlations between the expressions of MORC family genes, immune cell infiltration, TMB, MSI, and promoter methylation levels differed significantly, indicating that the MORC family genes fulfilled their functions through different mechanisms in different CRC phenotypes. In conclusion, our study revealed the mechanism of action of the MORC family genes in CRC, providing new targets for the prognosis and clinical management of CRC patients.

## AUTHORS’ CONTRIBUTIONS

The authors confirm their contribution to the paper as follows: Conception and design: B.H.L., C.F.Y.; administrative support: C.F.Y., L.B.C., H.C.; provision of study materials or patients: J.H.P.; collection and assembly of data: L.B.C., C.F.Y.; data analysis and interpretation: B.H.L., C.F.Y.; manuscript writing: All authors. All authors reviewed the results and approved the final version of the manuscript.

## Figures and Tables

**Fig. (1):**
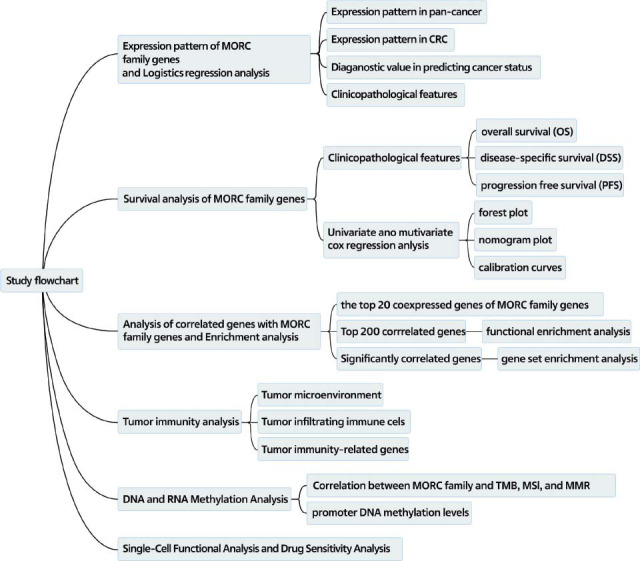
The flowchart of the study.

**Table 1:** The correlations between MORC family genes and cinical characteristics by logistic regression analysis.

Characteristics	Total(N)	MORC1 Odds Ratio(OR)	*P* value	Total(N)	MORC2 Odds Ratio(OR)	*P* value	Total(N)	MORC3 Odds Ratio(OR)	*P* value	Total(N)	MORC4 Odds Ratio(OR)	*P* value
T stage (T1&T2 *vs* T4&T3)	641	0.909 (0.152 - 5.440)	0.917	641	0.664 (0.450 - 0.979)	0.038	641	0.948 (0.646 - 1.392)	0.784	641	1.007 (0.686 - 1.479)	0.970
N stage (N0 *vs.* N1&N2)	640	0.624 (0.151 - 2.576)	0.514	640	0.673 (0.491 - 0.922)	0.014	640	1.214 (0.887 - 1.662)	0.226	640	1.673 (1.219 - 2.295)	0.001
M stage (M0 *vs.* M1)	564	0.519 (0.110 - 2.452)	0.408	564	0.586 (0.370 - 0.928)	0.023	564	1.160 (0.736 - 1.828)	0.523	564	1.782 (1.121 - 2.834)	0.015
Stage (Stage I&Stage II *vs.* Stage III&Stage IV)	623	0.667 (0.161 - 2.756)	0.576	623	0.632 (0.460 - 0.869)	0.005	623	1.225 (0.892 - 1.682)	0.209	623	1.739 (1.264 - 2.394)	< 0.001
Primary therapy outcome (PD&SD *vs.* PR&CR)	312	0.879 (0.084 - 9.161)	0.914	312	1.451 (0.734 - 2.870)	0.285	312	0.900 (0.456 - 1.775)	0.761	312	0.877 (0.445 - 1.728)	0.704
Gender (Female *vs.* Male)	644	0.189 (0.022 - 1.628)	0.129	644	0.940 (0.689 - 1.280)	0.693	644	0.872 (0.640 - 1.188)	0.385	644	0.988 (0.725 - 1.346)	0.937
Age (<= 65 *vs.* > 65)	644	0.515 (0.103 - 2.586)	0.420	644	1.754 (1.279 - 2.404)	< 0.001	644	1.322 (0.967 - 1.808)	0.080	644	0.927 (0.678 - 1.266)	0.633
Residual tumor (R0 *vs.* R1&R2)	510	1215.057 (0.003- NA)	0.287	510	0.677 (0.358 - 1.280)	0.230	510	2.677 (1.315 - 5.453)	0.007	510	2.052 (1.054 - 3.997)	0.035
CEA level (<= 5 *vs.* > 5)	415	0.224 (0.036-1.383)	0.107	415	0.797 (0.534 - 1.189)	0.266	415	1.078 (0.724 - 1.605)	0.712	415	0.928 (0.623 - 1.382)	0.712
Perineural invasion (No *vs.* Yes)	235	0.229 (0.005 - 10.443)	0.450	235	0.503 (0.257 - 0.984)	0.045	235	0.777 (0.409 - 1.479)	0.443	235	1.397 (0.776 - 2.517)	0.265
Lymphatic invasion (No *vs.* Yes)	582	1.406 (0.308 - 6.430)	0.660	582	0.972 (0.697 - 1.354)	0.866	582	2.179 (1.553 - 3.058)	< 0.001	582	1.731 (1.238 - 2.420)	0.001
History of colon polyps (No *vs.* Yes)	555	0.252 (0.050 - 1.279)	0.096	555	1.823 (1.268 - 2.622)	0.001	555	1.410 (0.984 - 2.020)	0.061	555	1.215 (0.850 - 1.737)	0.285

**Note:** The bold values means *p* value < 0.05, and the other unbold values mean *p* value > 0.05.

**Table 2:** The relationship between MORC4 and the clinicopathological data of 150 CRC patients.

Parameters	No. of Patients	Expression of MORC4		*P* value
		Low (57)	High (93)	
Gender	-	-	-	0.415
Male	84	34	50	-
Female	66	23	43	-
Age (years)	-	-	-	0.667
< 60	22	7	15	-
≥ 60	128	50	78	-
Size (maximal diameter)	-	-	-	0.145
>5 cm	52	17	35	-
≤ 5 cm	98	40	58	-
Differentiation	-	-	-	0.001
Well, moderate	126	53	73	-
Poor	24	4	20	-
TNM stage	-	-	-	0.005
I + II	89	42	47	-
III + IV	61	15	46	-
Tumor invasion	-	-	-	0.421
T1 + T2	16	9	7	-
T3 + T4	134	48	86	-
Lymph node status	-	-	-	0.012
Positive	58	15	43	-
Negative	92	42	50	-
Metastasis	-	-	-	0.001
M0	131	55	76	-
M1	19	2	17	-
Serum CEA (µg/L)	-	-	-	0.000
<5	70	40	30	-
≥5	80	17	63	-

## Data Availability

The data used in this study will be available from corresponding author upon request.

## LIST OF ABBREVIATIONS

AUC: Area Under Curve
CCK-8: Cell Counting Kit-8
CRC: Colorectal Cancer
MORC: Microrchidia
MMR: Mismatch Repair
MSI: Microsatellite Instability
NC: Negative Control
TMB: Tumor Mutation Burden
TME: Tumor Microenvironment

## References

[1] Chang J., Lin G., Ye M., Tong D., Zhao J., Zhu D., Yu Q., Zhang W., Li W. (2019). Decreased mean platelet volume predicts poor prognosis in metastatic colorectal cancer patients treated with first-line chemotherapy: Results from mCRC biomarker study.. BMC Cancer.

[2] Sun H., Wang H., Li X., Hao Y., Ling J., Wang H., Wang F., Xu F. (2023). Increased MAD2L2 expression predicts poor clinical outcome in colon adenocarcinoma.. Biocell.

[3] Nemati M., Rasmi Y., Rezaie J. (2023). Therapeutic application of mesenchymal stem cells-derived extracellular vesicles in colorectal cancer.. Biocell.

[4] Arnold M., Sierra M.S., Laversanne M., Soerjomataram I., Jemal A., Bray F. (2017). Global patterns and trends in colorectal cancer incidence and mortality.. Gut.

[5] Ruan Y., Lu G., Yu Y., Luo Y., Wu H., Shen Y., Gao Z., Shen Y., Cai Z., Li L. (2024). PF-04449913 inhibits proliferation and metastasis of colorectal cancer cells by down-regulating MMP9 expression through the ERK/p65 pathway.. Curr. Mol. Pharmacol..

[6] Manohar S.M., Joshi K.S. (2022). Promising anticancer activity of multitarget cyclin dependent kinase inhibitors against human colorectal carcinoma cells.. Curr. Mol. Pharmacol..

[7] Chen W., Zheng R., Baade P.D., Zhang S., Zeng H., Bray F., Jemal A., Yu X.Q., He J. (2016). Cancer statistics in China, 2015.. CA Cancer J. Clin..

[8] Wang H., Zhang L., Luo Q., Liu J., Wang G. (2021). MORC protein family-related signature within human disease and cancer.. Cell Death Dis..

[9] Zhang S., Guo A., Wang H., Liu J., Dong C., Ren J., Wang G. (2024). Oncogenic MORC2 in cancer development and beyond.. Genes Dis..

[10] Kim H., Yen L., Wongpalee S.P., Kirshner J.A., Mehta N., Xue Y., Johnston J.B., Burlingame A.L., Kim J.K., Loparo J.J., Jacobsen S.E. (2019). The gene-silencing protein MORC-1 topologically entraps DNA and forms multimeric assemblies to cause DNA compaction.. Mol. Cell.

[11] Li D.Q., Nair S.S., Kumar R. (2013). The MORC family.. Epigenetics.

[12] Pan Z., Ding Q., Guo Q., Guo Y., Wu L., Wu L., Tang M., Yu H., Zhou F. (2018). MORC2, a novel oncogene, is upregulated in liver cancer and contributes to proliferation, metastasis and chemoresistance.. Int. J. Oncol..

[13] Yang Z., Zhuang Q., Hu G., Geng S. (2019). MORC4 is a novel breast cancer oncogene regulated by miR-193b-3p.. J. Cell. Biochem..

[14] Liu M., Sun X., Shi S. (2018). MORC2 enhances tumor growth by promoting angiogenesis and tumor-associated macrophage recruitment *via* Wnt/β-catenin in lung cancer.. Cell. Physiol. Biochem.: Int. J. Experi. Cell. Physiol., Biochem., Pharmacol..

[15] Song Z., Yu J., Wang M., Shen W., Wang C., Lu T., Shan G., Dong G., Wang Y., Zhao J. (2023). CHDTEPDB: Transcriptome expression profile database and interactive analysis platform for congenital heart disease.. Congenit. Heart Dis..

[16] Liu W., Ye H., Liu Y.F., Xu C.Q., Zhong Y.X., Tian T., Ma S.W., Tao H., Li L., Xue L.C., He H.Q. (2018). Transcriptome-derived stromal and immune scores infer clinical outcomes of patients with cancer.. Oncol. Lett..

[17] Marabelle A., Fakih M., Lopez J., Shah M., Shapira-Frommer R., Nakagawa K., Chung H.C., Kindler H.L., Lopez-Martin J.A., Miller W.H., Italiano A., Kao S., Piha-Paul S.A., Delord J.P., McWilliams R.R., Fabrizio D.A., Aurora-Garg D., Xu L., Jin F., Norwood K., Bang Y.J. (2020). Association of tumour mutational burden with outcomes in patients with advanced solid tumours treated with pembrolizumab: Prospective biomarker analysis of the multicohort, open-label, phase 2 KEYNOTE-158 study.. Lancet Oncol..

[18] Nishiyama A., Nakanishi M. (2021). Navigating the DNA methylation landscape of cancer.. Trends Genet..

[19] Yuan H., Yan M., Zhang G., Liu W., Deng C., Liao G., Xu L., Luo T., Yan H., Long Z., Shi A., Zhao T., Xiao Y., Li X., Cancer S.E.A. (2019). CancerSEA: A cancer single-cell state atlas.. Nucleic Acids Res..

[20] Liu C.J., Hu F.F., Xie G.Y., Miao Y.R., Li X.W., Zeng Y., Guo A.Y. (2023). GSCA: An integrated platform for gene set cancer analysis at genomic, pharmacogenomic and immunogenomic levels.. Brief. Bioinform..

[21] Zhang Z., Pan Y., Zhao Y., Ren M., Li Y., Feng Y., Lu G., He S. (2022). Knockdown Wiskott-Aldrich syndrome protein family member 3 (WASF3) inhibits colorectal cancer metastasis and sensitizes to cisplatin through targeting ZNF471.. Biocell.

[22] Siegel R.L., Miller K.D., Goding Sauer A., Fedewa S.A., Butterly L.F., Anderson J.C., Cercek A., Smith R.A., Jemal A. (2020). Colorectal cancer statistics, 2020.. CA Cancer J. Clin..

[23] Takakura Y., Hinoi T., Egi H., Shimomura M., Adachi T., Saito Y., Tanimine N., Miguchi M., Ohdan H. (2013). Procalcitonin as a predictive marker for surgical site infection in elective colorectal cancer surgery.. Langenbecks Arch. Surg..

[24] Mulita F., Liolis E., Akinosoglou K., Tchabashvili L., Maroulis I., Kaplanis C., Vailas M., Panos G. (2022). Postoperative sepsis after colorectal surgery: A prospective single-center observational study and review of the literature.. Prz. Gastroenterol..

[25] Huh J.W., Lee W.Y., Park Y.A., Cho Y.B., Kim H.C., Yun S.H., Chun H.K. (2019). Oncological outcome of surgical site infection after colorectal cancer surgery.. Int. J. Colorectal Dis..

[26] Wang Y., Li X., Yu Y., Liang J. (2023). Risk factors for sepsis in patients with colorectal cancer complicated with gastrointestinal perforation and its impact on prognosis.. J. Gastrointest. Oncol..

[27] Klocker E.V., Barth D.A., Riedl J.M., Prinz F., Szkandera J., Schlick K., Kornprat P., Lackner K., Lindenmann J., Stöger H., Stotz M., Gerger A., Pichler M. (2020). Decreased activity of circulating butyrylcholinesterase in blood is an independent prognostic marker in pancreatic cancer patients.. Cancers (Basel).

[28] Dimopoulos M.P., Verras G.I., Mulita F. (2024). Editorial: Newest challenges and advances in the treatment of colorectal disorders; from predictive biomarkers to minimally invasive techniques.. Front. Surg..

[29] Coulter D.W., Boettner A.D., Kortylewicz Z.P., Enke S.P., Luther J.A., Verma V., Baranowska-Kortylewicz J. (2017). Butyrylcholinesterase as a blood biomarker in neuroblastoma.. J. Pediatr. Hematol. Oncol..

[30] Wang S., Huang X., Zhao S., Lv J., Li Y., Wang S., Guo J., Wang Y., Wang R., Zhang M., Qiu W. (2024). Progressions of the correlation between lipid metabolism and immune infiltration characteristics in gastric cancer and identification of BCHE as a potential biomarker.. Front. Immunol..

[31] Perry J., Zhao Y. (2003). The CW domain, a structural module shared amongst vertebrates, vertebrate-infecting parasites and higher plants.. Trends Biochem. Sci..

[32] Liang Y., Wu D., Qu Q., Li Z., Yin H. (2023). MORC4 plays a tumor-promoting role in colorectal cancer *via* regulating PCGF1/CDKN1A axis *in vitro* and *in vivo*.. Cancer Gene Ther..

[33] Zhao P., Ning J., Huang J., Wei B., Wang Z., Huang X. (2023). High expression of MORC2 is associated with poor clinical outcomes and immune infiltrates in colon adenocarcinoma.. Int. J. Gen. Med..

[34] Yi F.S., Zhai K., Shi H.Z. (2021). Helper T cells in malignant pleural effusion.. Cancer Lett..

[35] Meng Q., Zhao Y., Xu M., Wang P., Li J., Cui R., Fu W., Ding S. (2024). Increased circulating regulatory T cells and decreased follicular T helper cells are associated with colorectal carcinogenesis.. Front. Immunol..

[36] McGranahan N., Furness A.J.S., Rosenthal R., Ramskov S., Lyngaa R., Saini S.K., Jamal-Hanjani M., Wilson G.A., Birkbak N.J., Hiley C.T., Watkins T.B.K., Shafi S., Murugaesu N., Mitter R., Akarca A.U., Linares J., Marafioti T., Henry J.Y., Van Allen E.M., Miao D., Schilling B., Schadendorf D., Garraway L.A., Makarov V., Rizvi N.A., Snyder A., Hellmann M.D., Merghoub T., Wolchok J.D., Shukla S.A., Wu C.J., Peggs K.S., Chan T.A., Hadrup S.R., Quezada S.A., Swanton C. (2016). Clonal neoantigens elicit T cell immunoreactivity and sensitivity to immune checkpoint blockade.. Science.

[37] Marabelle A., Le D.T., Ascierto P.A., Di Giacomo A.M., De Jesus-Acosta A., Delord J.P., Geva R., Gottfried M., Penel N., Hansen A.R., Piha-Paul S.A., Doi T., Gao B., Chung H.C., Lopez-Martin J., Bang Y.J., Frommer R.S., Shah M., Ghori R., Joe A.K., Pruitt S.K., Diaz L.A. (2020). Efficacy of pembrolizumab in patients with noncolorectal high microsatellite instability/mismatch repair-deficient cancer: Results from the phase II KEYNOTE-158 study.. J. Clin. Oncol..

[38] Jones P.A. (2012). Functions of DNA methylation: Islands, start sites, gene bodies and beyond.. Nat. Rev. Genet..

[39] Deaton A.M., Bird A. (2011). CpG islands and the regulation of transcription.. Genes Dev..

[40] Mundorf A., Schmitz J., Güntürkün O., Freund N., Ocklenburg S. (2018). Methylation of MORC1: A possible biomarker for depression?. J. Psychiatr. Res..

[41] Wang T., Qin Z., Wen L., Guo Y., Liu Q., Lei Z., Pan W., Liu K., Wang X., Lai S., Sun W., Wei Y., Liu L., Guo L., Chen Y., Wang J., Xiao H., Bian X., Chen D., Wang B. (2018). Epigenetic restriction of Hippo signaling by MORC2 underlies stemness of hepatocellular carcinoma cells.. Cell Death Differ..

[42] Duan X., Guo G., Pei X., Wang X., Li L., Xiong Y., Qiu X. (2019). Baicalin inhibits cell viability, migration and invasion in breast cancer by regulating miR-338-3p and MORC4.. OncoTargets Ther..

