# Supplementary material for: Bioinformatics Analysis Reveals Microrchidia Family Genes as the Prognostic and Therapeutic Markers for Colorectal Cancer
Source: Endocr Metab Immune Disord Drug Targets. 2025 Jan 13;25(15):1240–61. doi: 10.2174/0118715303367767241231113110 (PMC12709531; doi:10.2174/0118715303367767241231113110)
Supplement: Supplementary file 1 [file EMIDDT-25-15-1240_SD1.pdf]

Supplementary Material

Bioinformatics Analysis Reveals Microrchidia Family Genes as the Prognostic and Therapeutic Markers for Colorectal Cancer

Binghui Liu[1, #], Lingbin Chen[1, #], Hui Chen[1, #], Juhua Pan[1, #] and Changfa Yu[2, *, #]

[1]Department of Pathology, Taizhou First People's Hospital, Huangyan Hospital of Wenzhou Medical University, Taizhou, Zhejiang, China; [2]Department of Laboratory Medicine, Taizhou First People's Hospital, Huangyan Hospital of Wenzhou Medical University, Taizhou, Zhejiang, China

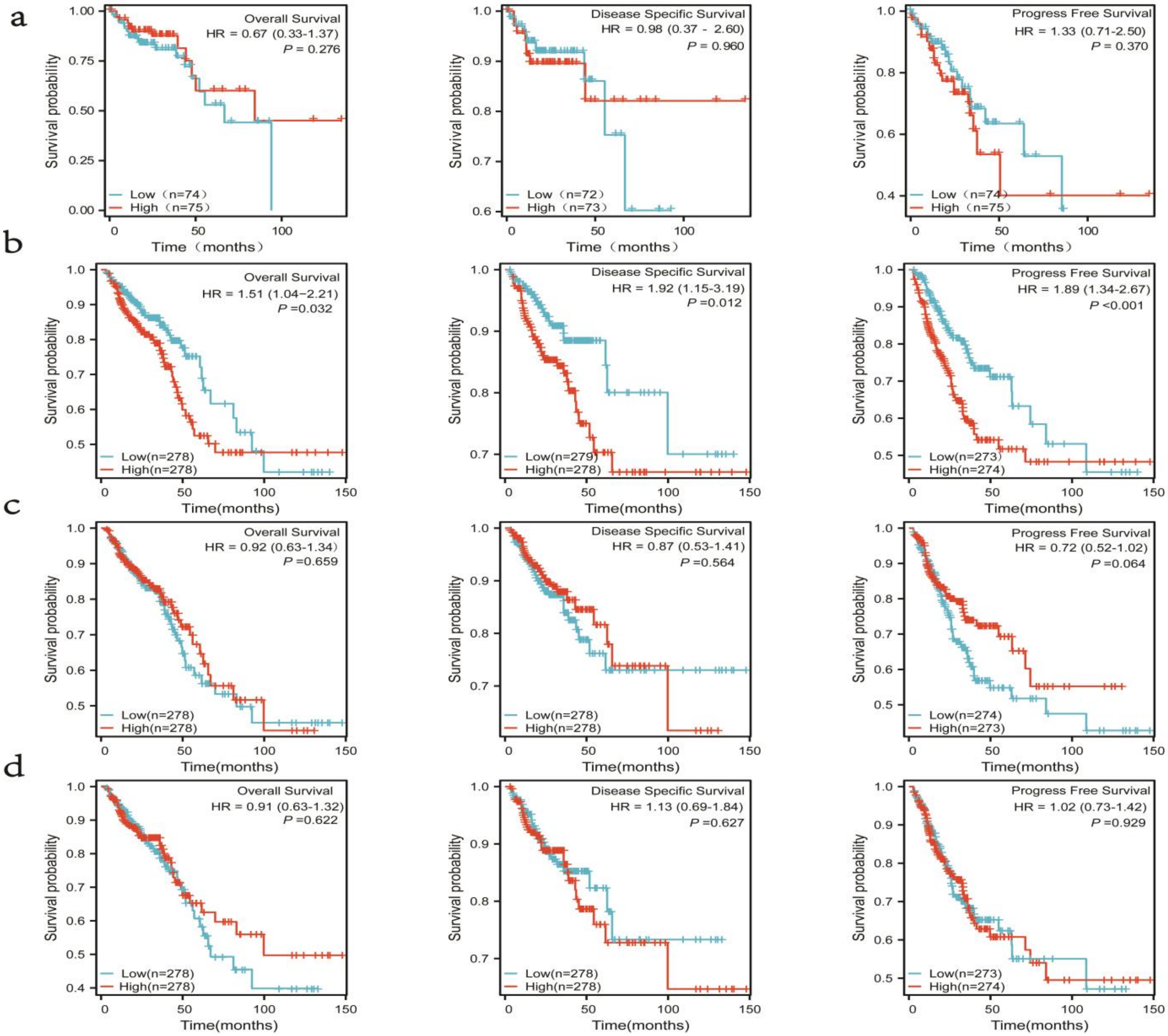

Fig. S1. The Kaplan Meier curves show the relationship between the expression of MORC family genes and overall survival (OS), disease-specific survival (DSS), and progression-free survival (PFS) in CRC patients, as determined by the Log-rank test; $P < 0.05$ was considered to be statistically significant. (**a**) MORC1, (**b**) MORC2, (**c**) MORC3, and (**d**) MORC4.

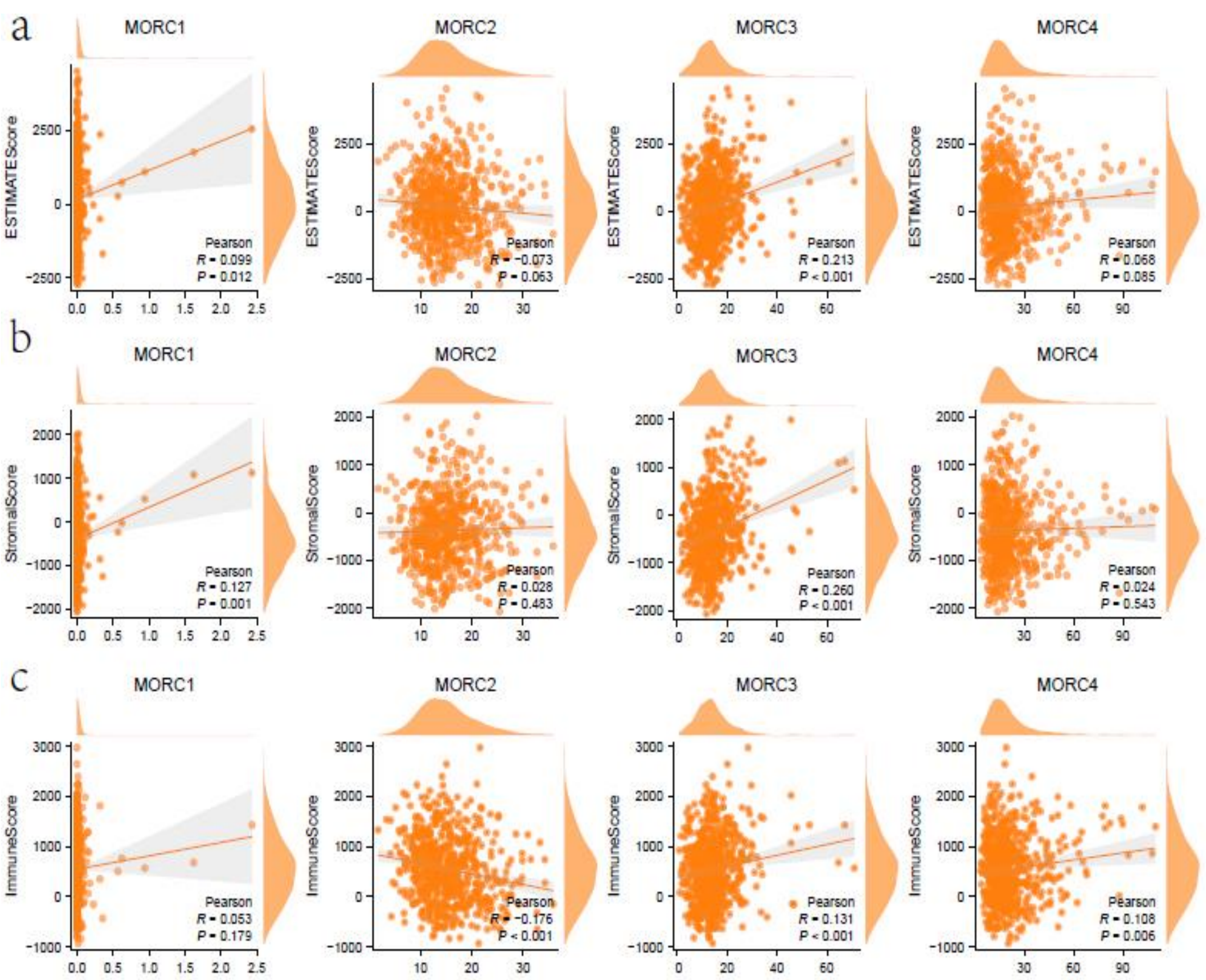

Fig. S2. The correlation between MORC family genes and the tumor microenvironment was determined by assessing the Estimate-Immune-Stromal score in CRC.

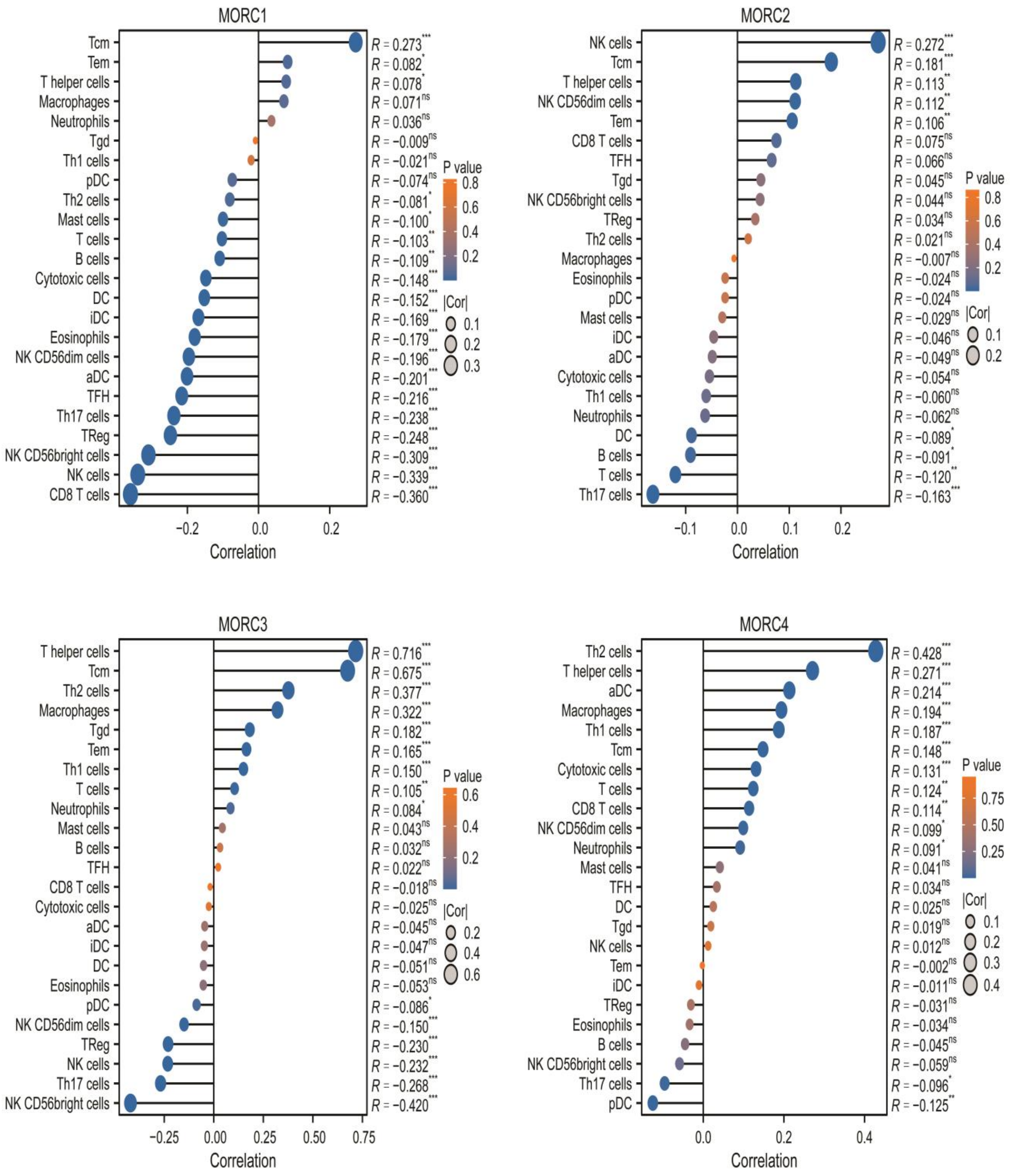

Fig. S3. Correlation between MORC family genes and immune cell infiltration in CRC.

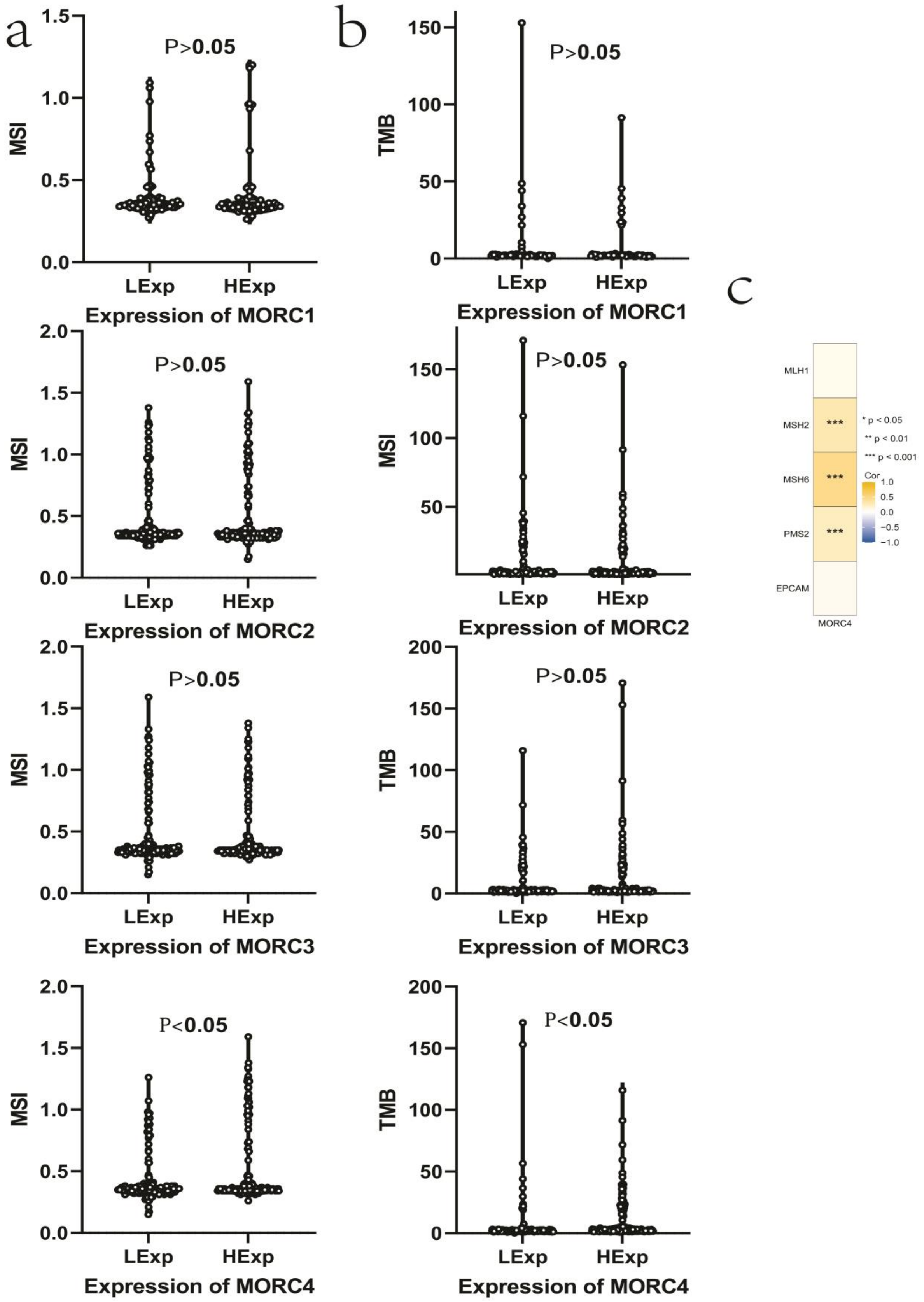

Fig. S4. The relationship between the high and low expression of MORC family genes and TMB (**a**) and MSI (**b**) in CRC. A correlation expression heatmap between MORC4 and MMRs (**c**).

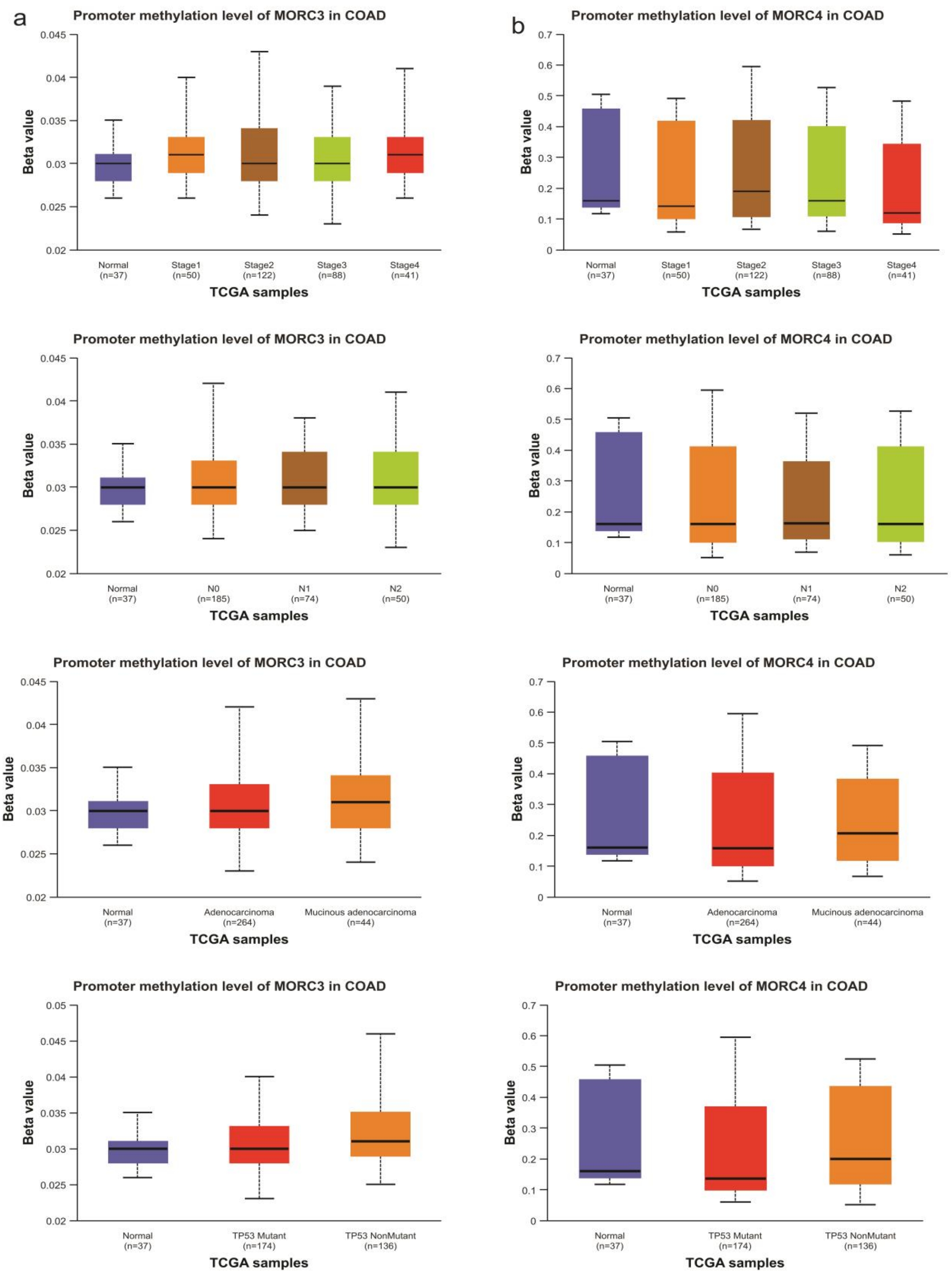

Fig. S5. The methylation level of MORC3 (**a**) and MORC4 (**b**) related to different clinicopathological features of CRC.
